# Personalised Medicine in shaping sustainable healthcare: a Delphi survey within the IC2PerMed project

**DOI:** 10.1093/eurpub/ckac129.429

**Published:** 2022-10-25

**Authors:** F Beccia, FA Causio, S Farina, C Savoia, T Osti, M Di Marcantonio, A Morsella, C Cadeddu, W Ricciardi, S Boccia

**Affiliations:** 1 Department of Life Sciences and Public Health, Università Cattolica del Sacro Cuore, Rome, Italy; 2 Faculty of Economics, Università Cattolica del Sacro Cuore, Rome, Italy; 3 Public Health Area, Fondazione Policlinico A. Gemelli IRCCS, Rome, Italy

## Abstract

**Background:**

Personalised medicine (PM) has the potential to transform health systems and make them more sustainable, by making the population healthier and allocating resources efficiently. European Union and China have become world leaders in the field of PM, increasing collaborations worldwide. In this context, the EU Commission in 2020 launched the IC2PerMed (Integrating China in the International Consortium for Personalised Medicine) project to provide key solutions to enable the convergence of European and Chinese stakeholders toward a common approach in PM.

**Methods:**

From a mapping exercise of policies and programs in PM in EU and China, we identified 20 priority items for shaping sustainable healthcare. Such items were submitted to several Chinese and European experts in PM involved in a 3-round Delphi survey. Experts were asked to review the items’ content and rate their validity and relevance on a 5-point Likert scale. Priorities reaching a Content Validity Index of more than 79% were included, between 70 and 79% were revised, and less than 70% were excluded.

**Results:**

Of 20 priorities submitted, 9 reached consensus. The priorities hinge on the resources allocation, defining in advance priority investment, and identifying new payment models for public reimbursement, health technology impact, and assessment importance, while integrating end-user perceptions into the whole innovation process. In addition, the pivotal role of multidisciplinary and cross-sectorial collaborations emerged. Ethical, legal, and social implications and the related costs should be always considered in policymaking, evaluation, and management of technological innovation.

**Conclusions:**

Integrating resources and setting a clear agenda for the implementation of PM would lead to a faster and more efficient translation into clinical practice. Developing policies valuing all the stakeholders’ contributions would implement PM adoption.

**Key messages:**

• Healthcare systems sustainability is a priority and PM could make the population healthier and help allocate resources more efficiently, hence reducing the overall costs of healthcare.

• The inter-sectoral collaborations in healthcare are fundamental to achieving the best standard of care. All stakeholders and policymakers should engage to foster sustainability.

